# Women's experiences of more than one termination of pregnancy within two years: a mixed‐methods study

**DOI:** 10.1111/1471-0528.14940

**Published:** 2017-10-20

**Authors:** C Purcell, J Riddell, A Brown, ST Cameron, C Melville, G Flett, Y Bhushan, L McDaid

**Affiliations:** ^1^ MRC/CSO Social and Public Health Sciences Unit University of Glasgow Glasgow UK; ^2^ NHS Greater Glasgow and Clyde Sexual and Reproductive Health Sandyford Central Glasgow UK; ^3^ Chalmers Centre NHS Lothian Edinburgh UK; ^4^ True Relationships & Reproductive Health Windsor QLD Australia; ^5^ NHS Grampian Sexual and Reproductive Health Aberdeen Community Health and Care Village Aberdeen UK; ^6^ NHS Tayside Gynaecology Assessment Unit Ninewells Hospital Dundee UK

**Keywords:** Abortion stigma, health services, intimate partner violence, mixed methods, repeat abortion, termination of pregnancy

## Abstract

**Objective:**

To examine the experiences of women seeking more than one termination of pregnancy (TOP) within 2 years.

**Design:**

Mixed methods study.

**Setting:**

Six TOP services across Scotland.

**Sample:**

Women presenting for TOP between July and December 2015.

**Methods:**

Descriptive and inferential analysis of quantitative survey data, thematic analysis of qualitative interview data and integrative analysis. In quantitative analysis, multinomial logistic regression was used to compare three groups: previous TOP within 2 years, previous TOP beyond 2 years and no previous TOP.

**Main outcome measures:**

Characteristics and experiences of women seeking TOP.

**Results:**

Of 1662 questionnaire respondents, 14.6% (*n* = 242) and 19.8% (*n* = 329) reported previous TOP within and beyond 2 years, respectively. The previous TOP within 2 years group was significantly less likely to own their accommodation than the no previous TOP group (adjusted odds ratio [aOR] 0.34, 95% CI: 0.18–0.62) and previous TOP beyond 2 years group (aOR: 0.44, 95% CI: 0.23–0.85); and more likely to report inconsistent (aOR: 1.63, 95% CI: 1.04–2.57; aOR: 1.95, 95% CI: 1.16–3.28) and consistent (aOR: 2.13, 95% CI: 1.39–3.26; aOR: 1.71, 95% CI: 1.07–2.76) contraceptive use than the no previous TOP and previous TOP within 2 years groups, respectively. Twenty‐three women from the previous TOP within 2 years group were interviewed. Qualitative and integrative analyses highlight issues relating to contraceptive challenges, intimate partner violence, life aspirations and socio‐economic disadvantage.

**Conclusions:**

Women undergoing more than one TOP within 2 years may experience particular challenges and vulnerabilities. Service provision should recognise this and move away from stigmatising discourses of ‘repeat abortion’.

**Funding:**

Scottish Government.

**Tweetable abstract:**

Women having two or more terminations of pregnancy in 2 years may face key challenges/vulnerabilities including intimate partner violence and socio‐economic disadvantage.

## Tweetable abstract

Women having two or more terminations of pregnancy in 2 years may face key challenges/vulnerabilities including intimate partner violence and socio‐economic disadvantage.

## Introduction

The fact that some women undergo more than one termination of pregnancy (TOP) is commonly framed as a concern for TOP provision, policy and research in the UK,^1^, ^2^ and globally.^3^, ^4^, ^5^ This interest may stem from associated concerns with: providing for those with unmet contraceptive needs; cost implications of TOP provision; potentially negative impacts on women of short pregnancy intervals; and drives toward patient‐centred care.^1^, ^6^ However, recent scholarship has focused on the problems of ‘repeat abortion’, and highlighted potentially discriminatory and stigmatising assumptions that underpin it.^7^, ^8^, ^9^

Factors found to be associated with more than one TOP are wide‐ranging and primarily relate to characteristics of women, contraceptive (non‐)use and broader contextual factors including deprivation and intimate partner violence (IPV), both in the UK^1^, ^2^, ^10^, ^11^ and globally.^4^, ^5^, ^12^, ^13^, ^14^, ^15^, ^16^, ^17^, ^18^ Some studies have proposed long‐acting reversible contraceptive (LARC) provision at TOP as an effective means of reducing the incidence of subsequent terminations.^18^, ^19^ Qualitative studies in this area have highlighted that women have unprotected sex for various reasons; that some struggle to effectively use preferred (typically user‐dependent) contraceptive methods; and that women experience each TOP differently, and as being the result of a unique set of circumstances including life stage, financial circumstances and relationship quality.^7^, ^20^ Despite provision and policy interest, there has been only limited research on this issue in the UK.

UK‐specific research has suggested that one‐third of women who had undergone two terminations had the second within 2 years of the first; and that around 60% did so within 5 years.^2^ However, few studies have specified the interval between terminations, and none to date have specifically focused on a fixed interval. Existing findings therefore present a partial picture, and do not address the complex, contextual specificities of women's reasons and experiences. The clinical experience of the authors combined with the TOP literature suggest that understanding the complexities of contextual detail – such as potential socio‐economic disadvantage – would probably contribute significantly to understanding why women seek more than one TOP in a relatively short window.

The objective of this study was therefore to produce a novel synthesis of qualitative and quantitative data, to draw out factors specific to women seeing more than one TOP in 2 years, and to interrogate these for commonalities and differences with any women seeking TOP.

## Methods

A mixed‐method design was devised to examine characteristics and experiences of women in Scotland seeking more than one TOP within 2 years. Quantitative and qualitative data were collected in parallel from NHS TOP assessment clinics in six participating NHS Health Board (administrative) areas from July to December 2015. Serving mixed urban and rural populations, these centres account for over 70% of ‘repeat’ terminations recorded in Scotland. Recruitment was facilitated by fully trained clinic staff, who were asked to circulate a questionnaire to all eligible women, and in‐depth interviews were conducted with women presenting at the same clinics who had undergone previous TOP in the preceding 2 years. Respondents were eligible if TOP was sought under Ground C of the 1967 Abortion Act, and they were: aged ≥16 years; able to read and speak enough English to enable participation; and able to provide informed consent**.**

### Quantitative survey

Eligible women were provided with an anonymous, self‐complete questionnaire to be returned to a drop‐box in clinic. A participant information sheet was provided with the questionnaire, and completion of the questionnaire was taken to indicate consent. The 31‐item questionnaire was based on pre‐validated questions adapted from existing sexual health surveys. Based on previous research^1^, ^2^, ^3^, ^4^, ^5^, ^7^, ^9^, ^12^, ^13^, ^14^, ^15^, ^16^, ^17^, ^18^, ^19^, ^20^, ^21^, ^22^ key measures identified were: age, education, ethnicity (due to small sample sizes within individual ethnicities, ethnicity was recoded into ‘white’ and ‘other’ – all those not reporting as White Scottish, British, Irish or any other White background), relationship status, deprivation quintile (by postcode),^22^ alcohol and tobacco consumption, and experience of IPV. Respondents provided information on TOP currently sought (estimated gestation [estimated because questionnaires were typically completed before confirmation of gestational age by ultrasound at clinic], main reason), previous TOP, and reproductive/contraceptive history.

Data were cross‐checked to assess response logic and analysed using stata 14 (Stata Corporation, College Station, TX, USA). Chi‐square tests were used for bivariate comparisons, and multinomial logistic regression was conducted to compare respondents reporting previous TOP within 2 years with those reporting previous TOP beyond the preceding 2 years and no previous TOP. The final model (adjusted for factors that were statistically significant at bivariate comparison *P* < 0.05) controlled for age and we report robust standard errors. As a sensitivity analysis, Health Board was included as a fixed effect. We compared previous TOP within 2 years with previous TOP beyond 2 years and, since we were also interested in differences between the two previous TOP groups, we re‐ran the model using no previous TOP as the reference group. We adjusted for factors significant at the bivariate level (*P *< 0.05) and report robust standard errors.

### Qualitative interviews

Women identified via routinely collected data as having undergone previous TOP in the preceding 2 years were approached in the consultation about interview participation at a later date. Written information was provided, and contact forms from those ‘opting‐in’ were passed to CP (study researcher) who made contact 2–3 weeks later to arrange an interview. We aimed to recruit a maximum of 40 women, although it was unknown how many eligible women would present.

Interviews were conducted up to 8 weeks following TOP, in a location of the woman's choosing or by telephone. CP obtained written consent from all participants before interviews. A flexible, semi‐structured topic guide was used to yield in‐depth personal accounts of women's experiences.^23^ Interviews addressed topics identified as relevant in previous research, including life circumstances, contraception and similarities/differences between terminations. Interviews lasted 60 minutes on average, and were digitally recorded, fully transcribed and anonymised. Participants received a £25 voucher as compensation for their time.

Data were analysed thematically, after repeated reading by CP in discussion with LM (study principal investigator), to compare interpretations, identify key themes, and develop a coding framework. Transcripts were coded and coded data sets were then further analysed for thematic linkages, and to explore similarities/differences across accounts. We used Nvivo 10 (QSR International 2012, Melbourne, Vic., Australia) to manage data.

### Integrative analysis

Following independent analyses, integration of both strands of data was undertaken. Key findings from each were positioned within a single matrix, which was subject to further interpretation by CP and LM, in consultation with all authors. In doing so, analysis addressed complementary findings from both strands and, via an iterative process, drew out key synergistic contributions.

## Results

### Sample characteristics

Complete questionnaires were collected from 1662 women, representing 38% of approximately 4415 women who underwent TOP via participating clinics in the recruitment period (statistics sourced from NHS Scotland Information Services Division [direct communication]). The mean age of respondents was 26.1 years (range 16–47, SD 6.4). Of all respondents, 82.6% (*n* = 1373) presented at ≤9 weeks of gestation (Table S1). More than half (60.8%, *n* = 1010) reported at least one previous pregnancy, and 47.2% (*n* = 784) had children. Approximately a quarter (72.2%, *n* = 1200) reported contraceptive use in the month before their most recent conception, although 31.5% (*n* = 524) indicated inconsistent use.

Almost half (44.5%, *n* = 739) of respondents lived in areas of highest relative deprivation^21^ and over half lived in rented accommodation (53.6%, *n* = 891). The majority reported post‐secondary education (69.3%) and ‘white’ ethnicity (92.3%, *n* = 1534). Over half reported minimal use of alcohol (56.3%, *n* = 936, ‘monthly or less’), and 41.9% (*n* = 696) reported tobacco use. Most were in a relationship/married (73.8%, *n* = 1227). A quarter (24.7%, *n* = 410) reported experience of IPV, with 27.8% (*n* = 114) of that group reporting IPV experienced in the preceding 12 months.

Fifty‐one ‘opt‐in’ forms were received, of which 23 were interviewed. The remainder withdrew or were uncontactable within a specified 6‐week timeframe. Of 23 women interviewed: 20 had undergone two terminations in 2 years; three had undergone three; 15 had undergone two terminations ever, while eight had three terminations or more. The mean age of interviewees was 25, and 13 had children (aged 1–16 years). Most lived in areas of highest relative deprivation (*n* = 14),^23^ and had post‐secondary education (*n* = 15). Fourteen were in a relationship/married, and nine were single.

### Comparing characteristics: previous TOP within 2 years, previous TOP beyond 2 years, and no previous TOP

Of all respondents, 34.4% (*n* = 571) reported previous TOP; 14.6% (*n* = 242) within the preceding 2 years (42.4% of those reporting any previous TOP). The majority (74.2%, *n* = 161) of those reporting previous TOP within 2 years gave their gestation at that termination as ≤9 weeks, and 4.9% (*n* = 12) at ≥14 weeks. A further 15.7% (*n* = 38) of the previous TOP within 2 years group gave the age of their youngest child as ≤2 years (a slightly higher figure than the 13.7% [*n* = 227] reported by the total sample). In the previous TOP within 2 years group, 87.2% (*n* = 211) reported discussing contraception with a health professional at previous TOP, and 19.9% of those (*n* = 42) had taken LARC (an implant, intrauterine device/system or injectable depot medroxyprogesterone acetate). There were statistically significant differences between the three groups regarding age, children, contraception, accommodation, education, alcohol and tobacco use, and IPV; but not in gestation, deprivation, ethnicity, or relationship status at most recent TOP.

The multinomial logistic regression models (Table 1) suggested that age, accommodation, contraceptive use and experience of IPV held a significant association with more than one TOP. The previous TOP within 2 years group was significantly less likely to own their accommodation than the previous TOP beyond 2 years group (adjusted OR [aOR] 0.44, 95% CI: 0.23–0.85) and no previous TOP group (aOR: 0.34, 95% CI: 0.18–0.62). The previous TOP within 2 years group was also significantly more likely to report contraception at most recent conception than the other two groups, though odds were slightly higher for the previous TOP within 2 years group regarding inconsistent use. The previous TOP beyond 2 years group was significantly more likely to report experience of IPV than the no previous TOP group (aOR: 1.44, 95% CI: 1.04–1.99). Those reporting previous TOP beyond 2 years were more likely than the no previous TOP group to report tobacco use (aOR: 1.40, 95% CI: 1.03–1.90), and were significantly less likely to report higher levels of alcohol use (twice weekly or more, aOR: 0.59, 95% CI: 0.37–0.94). No other factors were statistically significant.

**Table 1:** Multinomial logistic regression comparing respondents reporting previous TOP within 2 years, previous TOP beyond 2 years and no previous TOP (*n *= 1662), using age at most recent TOP as control

	Previous TOP within 2 years (*n* = 242) versus previous TOP beyond 2 years (*n* = 329)			Previous TOP within 2 years (*n* = 242) versus no previous TOP (*n* = 1091)			Previous TOP beyond 2 years (*n* = 329) versus no previous TOP (*n* = 1091)		
	aOR	95% CI	Robust SE	aOR	95% CI	Robust SE	aOR	95% CI	Robust SE
**Woman's age at most recent TOP (controlling factor)**	0.93^b^	0.90–0.97	0.02	1.02	0.99–1.05	0.02	1.09^b^	1.06–1.12	0.02
**Health Board**									
NHS Greater Glasgow and Clyde	1			1			1		
NHS Ayrshire and Arran	1.42	0.74–2.71	0.47	1.24	0.70–2.19	0.36	0.87	0.53–1.43	0.22
NHS Grampian	1.5	0.64–3.49	0.65	1.01	0.49–2.06	0.37	0.68	0.36–1.27	0.22
NHS Highland	1	0.45–2.22	0.41	0.75	0.37–1.54	0.28	0.75	0.43–1.33	0.22
NHS Lothian	1.77^b^	1.09–2.88	0.44	1.78^b^	1.16–2.74	0.39	1	0.69–1.46	0.19
NHS Tayside	1.51	0.84–2.74	0.46	1.27	0.75–2.13	0.34	0.84	0.53–1.32	0.2
**Children**									
No	1			1			1		
Yes	0.74	0.46–1.18	0.18	0.82	0.54–1.25	0.17	1.12	0.77–1.63	0.22
**Accommodation**									
Rented (private/social housing)	1			1			1		
Accommodation which I own	0.44^a^	0.23–0.85	0.15	0.34^b^	0.18–0.62	0.11	0.76	0.51–1.15	0.16
Accommodation which my family owns	0.67	0.40–1.14	0.18	0.69	0.45–1.07	0.15	1.03	0.67–1.57	0.22
Other	0.78	0.29–2.06	0.39	0.71	0.31–1.61	0.3	0.91	0.42–1.94	0.35
**Post‐secondary education**									
No	1			1			1		
Yes	0.79	0.52–1.20	0.17	0.79	0.55–1.13	0.15	1	0.71–1.39	0.17
**Alcohol use**									
Monthly or less	1			1			1		
Two to four times per month	1.16	0.75–1.78	0.26	1.17	0.80–1.72	0.23	1.01	0.72–1.43	0.18
Two or more times per week	1.11	0.60–2.07	0.35	0.66	0.38–1.13	0.18	0.59^a^	0.37–0.94	0.14
**Uses tobacco**									
No	1			1			1		
Yes	0.86	0.59–1.27	0.17	1.21	0.86–1.70	0.21	1.40^a^	1.03–1.90	0.22
**Contraception in month prior to most recent conception**									
Did not use	1			1			1		
Inconsistent use	1.95^a^	1.16–3.28	0.52	1.63^b^	1.04–2.57	0.38	0.84	0.57–1.24	0.17
Consistent use	1.71^a^	1.07–2.76	0.42	2.13^b^	1.39–3.26	0.46	1.24	0.87–1.76	0.22
**Experience of IPV**									
No	1			1			1		
Yes	1	0.67–1.49	0.21	1.43^c^	0.99–2.05	0.26	1.44^a^	1.04–1.99	0.24

a *P *< 0.01.

b *P *< 0.05.

c *P* value borderline significant.

### Experiences of more than one TOP within 2 years: integrative synthesis

This section synthesises quantitative findings outlined above with qualitative data generated in interviews with women who had undergone more than one TOP within 2 years. Qualitative data add nuance and point to key challenges relating to contraception, IPV and life aspirations. Integrative analysis suggests that these issues may be particularly acute among women seeking more than one TOP within 2 years. Table 2 presents data extracts illustrative of each point, and Table 3 presents the integrative matrix.

**Table 2:** Qualitative data by theme

Theme	Sub‐theme	Sample data extracts^a^
Contraceptive challenges	Most were using contraception at each conception	…every time I fell pregnant I've been on contraception. Every single time. […] that's why I was like, ‘There's no way I'm pregnant.’ But… **(CP: Was it the pill you'd been on each time or…?)** The pill twice, the coil once, and the patch once. (L07/21/TOP4)
	Feeling they had done all they could and yet became pregnant	[After first TOP] I went on the contraceptive pill. And […] I think just before [son] turned one, I found out I was pregnant again. I took the pill *and* I took the morning after pill. […] Obviously I didn't want to be pregnant again. And then after that termination I was actually on the jag [DMPA]. And I fell pregnant again. I just – it's hard to believe. (A02/24/TOP3)
	Partner role in contraception	I said to him that we need to be really careful and he's like: ‘Oh, no, nothing will happen’. So I took his word for it. And he didn't really want me to go on any contraceptive pills, so I was a bit reluctant and I didn't take anything and it happened again. […] [So] I was being more careful, but it just happened. The most recent time it happened because my mum wanted me to leave him. I told him and he was just like: ‘Oh, I'm definitely going to get you pregnant, I don't care’. (G06/27/TOP4)
	Self‐critical accounts of absence of effective contraception	It's [feelings of] guilt and shame, because…I shouldn't be doing it if I can't accept the responsibilities, like, the repercussions that come with having sex. I should have prevented it after what I went through [later TOP] last time. I was stupid and naive to think that it wouldn't happen to me again. I should never have put myself in the position where I could have fell pregnant again… (G01/27/TOP2)
	Negative attitudes of health professionals	[Doctor] said to me ‘You've been *extremely* unlucky, and there is a failure rate in the pill’ And I said ‘But three times?’ And she said that ‘life has a funny way of getting back at you’ and I might find in future I won't be able to have children because I've done this three times. And I was like… so… as in karma? […] At first I thought […] she does believe me, because my fear was that I was going to say [she had used contraception] and people were gonna be like ‘She's talking rubbish,’ y'know? And I felt, I kept, like, justifying myself. [… So] when she said *that* I was like ‘Oh my god’ […] That made me feel like, right, she's judging me a little bit… (H01/29/TOP3)
IPV		We had been together for about a year, we had our own place, we were at university, planning to get married […] But then our relationship started taking a strange turn. He was diagnosed with depression before I met him, so I knew about that. But there was this compulsive lying going on, there was strange things happening.[…] There was a couple of times that got physically violent. […] It ended up in a really bad argument, [a] fight which ended up with me having my knee slit open with a knife [and] a door smacked in my face, which chipped my teeth. (GG03/23/TOP2)
Life aspirations and socio‐economic precarity	Aspirations	[Partner] was like: ‘look, we're both clearly not ready. What have we achieved in the last six months? Nothing.’ Not in a bad way, he was like… **(CP: But what's different to…)** Yeah, what's different? Six months ago we both said that we would rather be home owners than renting. We both said that we'd like to be further up in our careers.(T03/22/TOP2)
	Relationship (in)stability	The first termination… the pregnancy wasn't to my husband… So, I'd never been pregnant before. My husband was away, he'd been away for some time. **(CP: Right… Does he work away, or…?)** Yeah… **(Uh huh. Ok.)** So I decided then that… I didn't want to keep the baby. […] I was a bit all over the place. I wasn't sure if I was leaving my husband or… what I was going to do. […] I wasn't really in a relationship with him, it was just something that kinda happened, the circumstances I was in, y'know? Turned my life upside down. (H03/38/TOP2)
	Concerns relating to existing caring responsibilities	My mum's got terminal cancer, so… there's a lotta stuff going on in my life with that, and I was helping my dad look after my grandparents. And my grandad only passed away three weeks ago. […] I've had a lot on my plate […] I was scared as well because, my other son, he only really goes away with his gran [for] maybe an hour on a Thursday. He's always with me. I don't really get any time to myself. […] Obviously I was thinking about my son as well, like, with money and stuff. ‘Cause we don't know when [partner's] going to get work and stuff like that. (A02/24/TOP3)
	Women's own health and wellbeing	My mood was slipping quite a lot and [I'd] been to the doctors [but] they couldn't find [antidepressant] that was working. So my most recent termination, I'd say it was kinda the hardest decision I had to make, because I wanted to keep it, but I had to kinda think of myself. I had to think of the kids I've got just now. […] We had finally found a tablet that was working for me, so [I] was a bit better mood‐wise, but I needed it upped. But they refused to do that ‘cause I was pregnant. So I was to suffer nine months with my mood the way it is? And hormones included in that? It would just have been horrendous. (A03/25/TOP3)

a Identifiers indicate participant number/age/total terminations.

**Table 3:** Quantitative and qualitative synthesis of data on women reporting previous TOP within 2 years

Key issues	Quantitative analysis (*n* = 242)	Qualitative analysis (*n* = 23)	Interpretation of synthesis
Contraceptive challenges	Majority reported contraceptive use before the most recent conception (80.2%) and previous TOP (72.2%). Compared with respondents seeking first TOP, the previous TOP <2 years (and >2 years) group was more likely to report contraceptive use, though use not necessarily consistent. 87.2% reported discussing contraception at previous TOP, although <20% of those chose LARC	Most described having tried various methods, including following previous TOP, and feeling they had tried to prevent pregnancy; attributed unintended conceptions to method failure. Many described feeling responsible for, and highly negative about, multiple unintended conceptions/terminations. Some described partner noncooperation with contraceptive use. Negative feelings compounded by perceived negative attitudes of health professionals	Women seeking more than one TOP <2 years are not treating TOP ‘like contraception’, and have tried various methods, which challenges common assumption. Relatively high post‐TOP contraceptive uptake did not prevent need for subsequent TOP; though improved LARC uptake may help to ameliorate this. Contracepting effectively can be challenging. With limited options, women should be supported not stigmatised
Experience of IPV	Previous TOP <2 years group was more likely than the no previous TOP group to report experience of IPV (borderline significant)	One‐third described experience of IPV, and suggested that this had been contributing factor in at least one TOP	Findings suggest IPV may be especially acute in <2 year context. Health professionals should be aware of this
Life aspirations and socio‐economic disadvantage	Aside from not wanting to be pregnant, most common reason for seeking each TOP in the <2 years group related to work/living circumstances and partner/family issues. Previous TOP <2 year group significantly less likely than previous TOP >2 years and no previous groups to live in accommodation they owned. Previous TOP <2 year group reported higher % than total sample of children aged ≤2 years. Small proportion cited own health and wellbeing as main reason for seeking TOP	Reasons relating to life aspirations and disadvantage included: not feeling ready; not established in own home; still being in full‐time education; relationship quality; financial (in)stability; concerns regarding balancing work/financial commitments/caring responsibilities. More than a third had experienced mental health problems. Reasons and circumstances at each tended to be different, and combined to create distinct experiences	Alongside contraception and IPV, this suggests women seeking more than one TOP may be experiencing specific (acute) vulnerabilities, and should be supported (regardless of whether vulnerabilities are reported). Not a ‘repeat’ of same circumstances/experiences

#### Contraceptive use

All interviewees cited method failure (*n* = 14) – of the pill, condoms and (for one) intrauterine device – or non‐use of contraception (*n* = 9) as the most immediate reason for unintended conceptions, which all the most recent pregnancies were presented as being. Women described difficulties with multiple methods of contraception, primarily relating to unwanted adverse effects. Those who had tried various hormonal methods (only four had never tried any) felt that they had done all they could, and yet experienced multiple unintended conceptions. Some participants related contraceptive non‐use to partner refusal/reluctance to use condoms, over which they had little influence, which speaks to a significant issue of control exercised by male partners. Despite these various factors, the absence of effective contraception was explained by women in highly self‐critical terms, as relating to their own failure or inaction. In many cases, these feelings were compounded by negative attitudes of health professionals with whom women had discussed their need for subsequent TOP.

The data synthesis suggests a complex overall picture regarding contraception. A key original finding is that the vast majority of women seeking more than one TOP within 2 years reported contraceptive use, and were more likely to do so than those seeking a first TOP, although use was not necessarily consistent. Effect sizes for both inconsistent and consistent use were similar in comparison of the previous TOP within 2 years/no previous TOP, and previous TOP within 2 years/previous TOP beyond 2 years groups, but differed from the no previous TOP/previous TOP beyond 2 years comparison. However, the 95% CI for the latter comparison shows some overlap with the preceding two comparisons (Table 1). That women had received contraceptive advice at previous TOP – and had tried a range of methods, but encountered problems culminating in further unintended conceptions – highlights difficulties faced by women as they try to use contraception effectively. These findings suggest that women seeking more than one TOP are not treating TOP ‘like contraception’, as is often assumed, but that TOP offers an essential alternative for those who experience contraceptive difficulties.

#### IPV

Qualitative data echoed quantitative findings regarding an association between more than one TOP and IPV (Table S1). A third of interviewees had experienced IPV at some time, and described this as a contributing factor in at least one TOP. Several were in relationships that had not initially appeared to them to be abusive, and had terminated relatively ‘planned’ pregnancies after violence escalated. One participant explained discovering that she was pregnant shortly after an instance of physical violence. She struggled to decide what was best, concluding: ‘It was only the fact that he was a psycho[path] that was going to impinge on it. I'd have just felt really too much guilt if I brought a child into that environment’. Her account of this issue is emblematic of ways in which participants took responsibility for the decision – including responsibility for protecting a potential child from violence – even when circumstances were out with their control. In the quantitative data, effect sizes for IPV were similar in the previous TOP within 2 years/no previous TOP and previous TOP beyond 2 years comparisons, but differed in the previous TOP within 2 years/previous TOP beyond 2 years comparison (1.4 versus 1.00); although the CI in this comparison shows some overlap with the former two. Analytic synthesis here suggests that IPV is a key issue which may be especially acute for women seeking more than one TOP within 2 years.

#### Life aspirations

**Figure 1:**
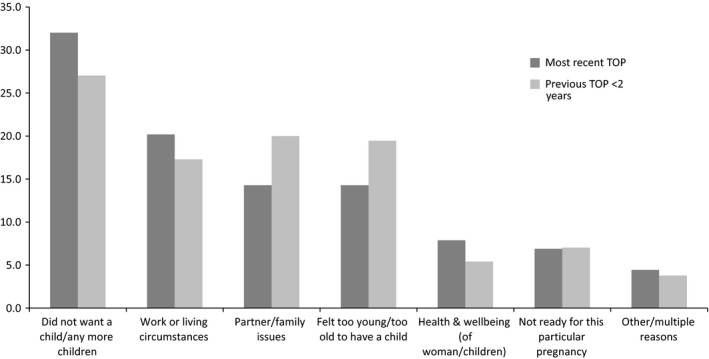
Main reason for seeking TOP at current and previous TOP within 2 years (*n* = 242).

The qualitative data build a more complex picture of women's reasons for seeking more than one TOP in 2 years that relates specifically to their life aspirations. Reasons for each TOP echoed those identified in the quantitative analysis (illustrated in Figure 1), and included: not feeling ready; not wanting a/another child at that time, or at all; not owning or being established in their own home; still being in full‐time education; and hopes/ambitions, career‐related or otherwise.

Interpretive synthesis highlights one further cluster of issues, around life aspirations and socio‐economic disadvantage experienced by women seeking more than one TOP. The picture presented by our analysis speaks to the interrelationship between women's financial and caring commitments, the latter being typically unpaid and disproportionately falling to women. It also fleshes out understanding of other factors in women's decision‐making, such as relationship quality; financial (in)stability; balancing work commitments; and concerns regarding the impact of the pregnancy on the time, attention and other (including emotional) resources available to their existing children, or others for whom they had caring responsibilities. Integrative analysis also foregrounds that contextual difficulties were compounded for some women by their having experienced unplanned pregnancies at a short interval after childbearing, and/or by concerns for their own mental or physical health. This was most clearly apparent in the fact that more than a third of the women interviewed had experienced mental health problems such as depression and anxiety.

Crucially, although many of the experiences and reasons reported across our data are familiar, and echo those of any women seeking TOP, the majority of interview participants described their reasons and circumstances *at each TOP* as being, to them, quite distinct, and not repeats of the same. Each was experienced in relatively unique ways, which highlights the complexities of women's lives.

It's crazy to think how different they were actually…I can't really think about anything that was similar to be honest. They just seem totally – from how I felt about it…to the actual procedure itself – felt totally different.

(G02/23/TOP2) [Identifier gives participant number/age/total terminations]

## Discussion

### Main findings

Our study has identified key differences between women seeking more than one TOP within 2 years and those reporting no previous TOP, or previous TOP beyond the preceding 2 years. It has brought to the fore issues that suggest that it is appropriate to reframe these differences as relating less to behaviours or characteristics per se, and more to particular challenges or vulnerabilities, underpinned by gender and socio‐economic inequalities. As we identify in our analytic synthesis, these related particularly to contraceptive difficulties, IPV and socio‐economic disadvantage. We therefore address a significant knowledge gap, to more effectively inform TOP policy and provision, and give weight to the critique of a potentially stigmatising policy and provide focus on ‘repeat abortion’.

### Strengths and limitations

A notable strength of this study is its originality of focus, being the first research to address women's experiences of more than one TOP in a fixed time frame, and the first to use a mixed‐methods approach to capture breadth and specificity in relation to these experiences. Our interpretive synthesis presents a robust picture of the contextual complexities underpinning women's need for more than one TOP. A limitation is that the study was cross‐sectional, and respondents who had not previously undergone TOP may do so again in future. However, a methodology that would facilitate comparison between women who had undergone only one and more than one TOP across their lifetime would introduce significant issues such as recall bias and attrition. Another limitation is that recruiting staff were unable to record how many women declined to participate, meaning that our response rate is estimated.

### Interpretation (in light of other evidence)

On the whole, our findings echo existing research that found associations between more than one TOP and factors including age, contraceptive challenges, accommodation and IPV.^1^, ^2^, ^3^, ^4^, ^5^, ^10^, ^11^, ^12^, ^13^, ^14^, ^15^, ^16^, ^17^ They also evidence the suggestion that women experience terminations quite differently, and not as ‘repeats’ of the same, rendering the shorthand of ‘repeat abortion’ misleading. A striking feature of our interpretive synthesis is its identification of a range of potential challenges or vulnerabilities may be experienced by women in this position.

The fact that some women continued not to use effective contraception following previous TOP also speaks to the complexities of why women have unprotected sex even when aware of risk.^21^ Furthermore, that many were *not* using more reliable LARC methods suggests that LARC may continue to offer an essential option to women who find user‐controlled methods challenging. However, the significance of unwanted perceived adverse effects also highlights the need to address what women do (not) find acceptable,^24^ and to develop more sophisticated, acceptable contraceptive methods.

The interpretive synthesis also underlines the established association between IPV and TOP, particularly more than one.^25^, ^26^ Our analysis suggests that this should be a key concern in the Scottish context. Many frontline providers are already acutely aware of the association between TOP and IPV. However, we suggest that more could be done – within services and regarding perceptions of TOP more broadly – to recognise that women seeking more than one TOP at relatively short intervals may be experiencing acute difficulties in this respect and/or have experienced IPV in the past.

Our findings highlight not only the role of relationship stability and factors beyond women's control – which have major relevance to the continuation of a pregnancy^27^ – but also the salience of mental wellbeing to women's feelings about the feasibility of a pregnancy. These findings underscore the essential role of accessible TOP in enabling women to achieve their aspirations, and to safeguard their mental health. It may also suggest emerging issues relating to a disjuncture between the limitations increasingly faced by younger women in the current political and economic climate, versus socio‐economic norms/expectations (e.g. regarding home ownership) transmitted from older generations.

Lastly, we note that we do not flag potential ‘vulnerabilities’ with the aim of victimising all women seeking more than one TOP, particularly as this may also be experienced as empowering. It is imperative that the organisation of TOP provision – in practical terms, and regarding the ethos of services – gives equal credence to the challenging circumstances of many women's lives, women's moral agency, and the need for all women to be supported in ending pregnancies that they do not feel able or want to continue.

## Conclusions

This study presents a holistic picture of women's experiences of seeking more than one TOP within 2 years, and highlights key factors including potentially acute challenges and vulnerabilities that women face regarding contraception, IPV and socio‐economic disadvantage. These challenges and vulnerabilities suggest that, in clinical practice, women seeking subsequent TOP should be approached with awareness and empathy. Providers should be supported in doing so, including through the provision of more extensive training around IPV. In terms of policy and patient‐centred care, there is a tangible need to effectively (re)position TOP as an acceptable and essential option on the spectrum of reproductive control. A focus should be maintained on improving access to contraceptive methods that are not only reliable but also right for, and acceptable to, individual women. On the whole, our findings add weight to evidence suggesting that policy and provision approaches, which focus specifically on women seeking more than one TOP, should be reconsidered. Stigmatising and misleading discourses of ‘repeat abortion’ should be rejected.

### Disclosure of interests

None declared. Completed disclosure of interests form available to view online as supporting information.

### Contribution to authorship

LM devised the study, CP and LM devised the paper, and CP wrote the first draft. JR conducted quantitative analysis in consultation with LM and contributed to manuscript drafts. CP conducted qualitative analysis in consultation with LM, who also advised on integration of findings. All authors – including SC, CM, GF, AB and YB – contributed to interpretation of the data by commenting on successive drafts, and approved the final version.

### Details of ethics approval

Ethical approval was obtained from the NHS West of Scotland Research Ethics Committee 5 (15/WS/0076, approved 29/04/2017), and NHS Greater Glasgow and Clyde Research and Development Office.

### Funding

The WEMA Study was commissioned and funded by the Scottish Government. Lisa McDaid and Julie Riddell are funded by the UK Medical Research Council (MRC) and Scottish Government Chief Scientist Office (CSO) at the MRC/CSO Social and Public Health Sciences Unit, University of Glasgow (MC_UU_12017/2, MC_UU_12017/11, SPHSU11). Open access was funded by the MRC/CSO SPHSU, University of Glasgow.

## Supporting information


**Table S1.** Quantitative sample characteristics for respondents reporting previous TOP <2 years, previous TOP >2 years, or no previous TOP (*n* = 1662).
