# Supplementary material for: Women's experiences of more than one termination of pregnancy within two years: a mixed‐methods study
Source: BJOG. 2017 Oct 20;124(13):1983–92. doi: 10.1111/1471-0528.14940 (PMC5725730; doi:10.1111/1471-0528.14940)
Supplement: Supplementary file 1 — Table S1. Quantitative sample characteristics for respondents reporting previous TOP <2 years, previous TOP >2 years, or no previous TOP (n = 1662). [file BJO-124-1983-s001.pdf]

Table S1. Quantitative sample characteristics for respondents reporting previous TOP <2 years, previous TOP >2 years, or no previous TOP (n= 1662)

		Previous TOP <2 years (n=242)	Previous TOP >2 years (n=329)	No previous TOP (n=1091)	Total (1662)	Chi-square p-value
		N *(%)*	N *(%)*	N *(%)*	N *(%)*	
**Health Board**	**NHS Ayrshire and Arran**	28 *(11.6)*	38 *(11.6)*	108 *(9.9)*	174 *(10.5)*	0.310
	**NHS Grampian**	15 *(6.2)*	21 *(6.4)*	88 *(8.1)*	124 *(7.5)*	
	**NHS Greater Glasgow and Clyde**	53 *(21.9)*	100 *(30.4)*	278 *(25.5)*	431 *(25.9)*	
	**NHS Highland**	16 *(6.6)*	26 *(7.9)*	100 *(9.2)*	142 *(8.5)*	
	**NHS Lothian**	88 *(36.4)*	97 *(29.5)*	339 *(31.1)*	524 *(31.5)*	
	**NHS Tayside**	42 *(17.4)*	47 *(14.3)*	178 *(16.3)*	267 *(16.1)*	
**Woman's age at most recent presentation for TOP**	**<20**	24 *(9.9)*	6 *(1.8)*	220 *(20.2)*	250 *(15.0)*	<0.001
	**20-24**	85 *(35.1)*	65 *(19.8)*	366 *(33.5)*	516 *(31.0)*	
	**25-29**	71 *(29.3*	106 *(32.2)*	231 *(21.2)*	408 *(24.5)*	
	**30-34**	39 *(16.1)*	81 *(24.6)*	158 *(14.5)*	278 *(16.7)*	
	**35+**	23 *(9.5)*	71 *(21.6)*	116 *(10.6)*	210 *(12.6)*	
**Children**	**No**	100 *(41.7)*	95 *(29.4)*	325 *(43.8)*	520 *(31.3)*	<0.001
	**Yes**	140 *(58.3)*	228 *(70.6)*	417 *(56.2)*	785 *(47.2)*	
**Contraception in month prior to most recent conception**	**Did not use**	47 *(19.8)*	88 *(27.5)*	305 *(27.6)*	440 *(26.5)*	<0.01
	**Inconsistent use***	75 *(31.6)*	86 *(26.9)*	386 *(37.9)*	524 *(31.5)*	
	**Always used**	115 *(48.5)*	146 *(45.6)*	415 *(37.5)*	676 *(40.7)*	
**Deprivation (SIMD quintile)**	**1 and 2 (most deprived)**	116 *(50.4)*	147 *(46.4)*	476 *(46.2)*	739 *(44.5)*	0.805
	**3**	36 *(15.7)*	58 *(18.3)*	187 *(18.1)*	281 *(16.9)*	
	**4 and 5 (least deprived)**	78 *(33.9)*	112 *(35.3)*	368 *(35.7)*	558 *(33.6)*	
**Accommodation**	**Rented (private/ social housing)**	161 *(67.1)*	200 *(61.2)*	530 *(48.9)*	891 *(53.6)*	<0.001
	**Accommodation which I own**	18 *(7.5)*	64 *(19.6)*	172 *(15.9)*	254 *(15.3)*	
	**Accommodation which parents/ family own**	53 *(22.1)*	52 *(15.9)*	318 *(29.4)*	423 *(25.5)*	
	**Other**	8 *(3.3)*	11 *(3.4)*	63 *(5.8)*	82 *(4.9)*	

**Post-secondary education**	**No**	87 *(37.5)*	102 *(31.4)*	277 *(26.1)*	466 *(28.0)*	0.001
	**Yes**	145 *(62.5)*	223 *(68.6)*	784 *(73.9)*	1152 *(69.3)*	
**Ethnicity**	**White**	218 *(91.6)*	310 *(94.8)*	1006 *(92.9)*	1534 *(92.3)*	0.304
	**Other°**	20 *(8.4)*	17 *(5.2)*	77 *(7.1)*	114 *(6.9)*	
**Alcohol use**	**Monthly or less**	149 *(63.7)*	197 *(62.1)*	590 *(56.2)*	936 *(56.3)*	<0.05
	**2-4 times per month**	61 *(26.1)*	81 *(25.6)*	279 *(26.6)*	421 *(25.3)*	
	**2+ times per week**	24 *(10.3)*	39 *(12.3)*	181 *(17.2)*	244 *(14.7)*	
**Tobacco use**	**No**	120 *(50.0)*	161 *(49.2)*	671 *(62.1)*	952 *(57.3)*	<0.001
	**Yes**	120 *(50.0)*	166 *(50.8)*	410 *(37.9)*	696 *(41.9)*	
**Relationship status**	**Single**	47 *(19.7)*	82 *(25.2)*	288 *(26.6)*	417 *(25.1)*	0.086
	**In relationship/Married/Other**	191 *(80.3)*	243 *(74.8)*	793 *(73.4)*	1227 *(73.8)*	
**Experience of IPV**	**No**	158 *(69.3)*	217 *(68.0)*	813 *(77.4)*	1188 *(71.5)*	0.001
	**Yes**	70 *(30.7)*	102 *(32.0)*	238 *(22.6)*	410 *(24.7)*	
**IPV breakdown**						
**Afraid of a partner**	**No**	171 *(70.7)*	237 *(72.0)*	895 *(82.0)*	1303 *(78.4)*	<0.001
	**Yes**	53 *(21.9)*	78 *(23.7)*	145 *(13.3)*	276 *(16.6)*	
**Told by a partner who you could see and where you could go**	**No**	167 *(69.0)*	245 *(74.5)*	886 *(81.2)*	1298 *(78.1)*	**<0.001**
	**Yes**	55 *(22.7)*	71 *(21.6)*	151 *(13.8)*	277 *(16.7)*	
**Humiliated or emotionally abused in other ways by a partner**	**No**	162 *(66.9)*	231 *(70.2)*	881 *(80.8)*	1274 *(76.7)*	**<0.001**
	**Yes**	60 *(24.8)*	81 *(24.6)*	156 *(14.3)*	297 *(17.9)*	
**Non-consensually physically hurt by a partner**	**No**	170 *(70.2)*	236 *(71.7)*	914 *(83.8)*	1320 *(79.4)*	**<0.001**
	**Yes**	51 *(21.1)*	78 *(23.7)*	120 *(11.0)*	249 *(15.0)*	
**Forced by a partner to have any sexual activity**	**No**	201 *(83.1)*	284 *(86.3)*	981 *(89.9)*	1466 *(88.2)*	**<0.01**
	**Yes**	23 *(9.5)*	29 *(8.8)*	57 *(5.2)*	109 *(6.6)*	

*'Inconsistent use': where respondents indicated that they had not used contraception on every occasion, or had been aware of method failure on one or more occasions.

° Due to small sample sizes within individual ethnicities, ethnicity was recoded into 'white' and 'other' (all those not reporting as White Scottish, British, Irish or any other White background).]
